# Prolonged Shedding of SARS-CoV-2 in Feces of COVID-19 Positive Patients: Trends in Genomic Variation in First and Second Wave

**DOI:** 10.3389/fmed.2022.835168

**Published:** 2022-03-15

**Authors:** Mallika Lavania, Madhuri S. Joshi, Sujata S. Ranshing, Varsha A. Potdar, Manohar Shinde, Nutan Chavan, Santosh M. Jadhav, Prasad Sarkale, Sreelekshmy Mohandas, Pradeep M. Sawant, Sanjaykumar Tikute, Vikram Padbidri, Sampada Patwardhan, Rohan Kate

**Affiliations:** ^1^Enteric Viruses Group, ICMR-National Institute of Virology, Pune, India; ^2^National Influenza Centre, ICMR-National Institute of Virology, Pune, India; ^3^Bioinformatics and Data Management Group, ICMR-National Institute of Virology, Pune, India; ^4^Microbial Containment Laboratory, ICMR-National Institute of Virology, Pune, India; ^5^Microbiology and Infection Control Jehangir Hospital, Pune, India; ^6^Microbiology and Hospital Infection Control, Deenanath Mangeshkar Hospital and Research Center, Pune, India; ^7^Department of Medicine, Lokmanya Hospital Chinchwad, Pune, India

**Keywords:** SARS-CoV-2, COVID-19, fecal, real time RT-PCR, NGS

## Abstract

The main route of the transmission of the severe acute respiratory syndrome coronavirus 2 (SARS-CoV-2) are through respiratory pathways and close contact of human-to-human. While information about other modes of transmission is comparatively less, some published literature supporting the likelihood of a fecal-oral mode of transmission has been accumulating. The diagnosis of SARS-COV-2 infected cases is based on the real-time reverse transcription-PCR (RT-PCR). The fecal excretion of SARS-COV-2 has been reported frequently, however, the role of fecal viral load with the severity of disease is not yet clear. Our study focused on the investigation of SARS-CoV-2 shedding in the fecal samples of patients with coronavirus disease 2019 (COVID-19). A total of 280 RT-PCR-positive patients were enrolled, among them 15.4% had gastrointestinal (GI) symptoms. It was shown that 62% of the patients were positive for SARS-CoV-2 RNA in fecal specimens. This positivity was not related to the presence of GI symptoms and the severity of disease. The next generation sequencing [NGS] of SARS-CoV-2 from fecal samples of patients was performed to analyze mutational variations. Findings from this study not only emphasized the potential presence of SARS-CoV-2 in feces, but also its continuing mutational changes and its possible role in fecal-oral transmission.

## Introduction

Since December 2019, several cases of pneumonia have been reported globally. Pneumonia-associated respiratory syndrome is causing due to this sever acute respiratory syndrome coronavirus 2 (SARS-CoV-2) across the globe. This positive-sense single-stranded RNA virus can cause a wide range of symptoms in infected persons which includes problem in breathing, dry cough, fever, and diarrhea. The clinical spectrum ranged from asymptomatic or mild respiratory tract infection to severe pneumonia with acute respiratory distress syndrome or multiorgan failure leading to a fatal outcome (1, 2).

In the previous SARS outbreak (2002–2003), 16–73% of patients had a symptom of diarrhea during the course of the disease, mainly within the first week of illness (3, 4). RNA from the SARS-CoV-2 was detected in fecal only from the 5th day of illness onward, and the proportion of fecal specimens positive for viral RNA progressively increased and reached on maximum at the 11th day of the illness, a small proportion of patients showed the presence of viral RNA even after 30 days of illness (5). The process for gastrointestinal (GI) tract infection of SARS-CoV-2 is proposed to be the angiotensin-converting enzyme 2 (ACE2) cell receptor. There are published reports that viral RNA can be detected in clinical specimens, such as oral swabs, sputum, feces, urine, and tears of positive patients with coronavirus disease 2019 (COVID-19) (6–8). Genomes of RNA infections transform rapidly and go through fast development, which could eventually influence their infectivity and contagiousness. Observing of changes inside the genome of SARS-CoV-2 at the community level is critical for following the outbreak situations, following the transmission chains and understanding the evolution of virus (9). Be that as it may, there are still difficulties in getting a good quality SARS-CoV-2 genome straight forward from clinical examples, particularly for those with low viral load.

This study focused on the detection of SARS-CoV-2 infection, which subsequently trigger the development of various clinical symptoms from fecal specimens besides respiratory specimens. Along with this, we aimed to isolate the virus from SARS-CoV-2-positive samples to work out viral infectivity.

## Materials and Methods

### Study Design and Patients

This was a multi-center study. The enrolled patients were admitted to Deenanath Mangeshkar, Jehangir, Lokmaanya hospitals Pune, Western India from May 2020 to August 2021. All the registered patients tested positive for SARS-CoV-2 RNA in oro/nasopharyngeal swab specimens by real-time reverse transcription PCR (RT-PCR).

### Specimen Collection From Patients With COVID-19

In this study, 280 patients were recruited. Fecal samples were collected from a laboratory confirmed patients with COVID-19 (throat swab/nasal swab). All the fecal samples collected from the hospitals, transported to Indian Council of Medical Research (ICMR)-National Institute of Virology, Pune and stored at −20°C until SARS-CoV-2 testing. To avoid contamination, all types of samples must be transported in sterile containers. While collecting the sample, workers should wear protective equipment, such as disposable gloves, N-95 mask, solid front or wrap-around gowns with sleeves that fully cover the forearms, head coverings, shoe covers, and face shield. The fecal specimens of positive patients with COVID-19 were collected at two time points during the hospitalization and at the time of discharge, and tested for SARS-CoV-2 RNA by RT-PCR. Patients found positive at the time of discharge were further followed-up for testing on monthly basis.

### Extraction of RNA From Fecal Specimens

Approximately 30% fecal suspensions in 0.01 M phosphate buffered saline (PBS), pH 7.4 were prepared by centrifuging the suspensions at 4,000 rpm (Hettich Universal 320R centrifuge) for 10 min to remove the debris. The viral RNA was extracted from 30% (w/v) suspensions of fecal specimens using spin columns Qiagen Viral RNA extraction Kit (Qiagen, Hilden, Germany) as per manufacturer's instructions.

### RTPCR and Determination of the Copy Number

The detection of SARS-CoV-2 viral RNA (which genes) from the fecal specimen was done in accordance with the protocol described earlier (10). A 25-μl reaction was set up containing 10 μl of RNA extracted from fecal samples, 12.5 μl of 2 X reaction buffer provided with the Superscript III one step RT-PCR system with Platinum Taq Polymerase (Invitrogen), 0.5 μl of reverse transcriptase/Taq mixture from the kit, and 1.5 μl of primer probe mix for each reaction. Thermal cycling was performed at 55°C for 10 min for reverse transcription, followed by 95°C for 3 min and then 45 cycles of 95°C for 15 s, 58°C for 30 s.

A standard curve of RNA generated from SARS-CoV-2 of known titer was used to quantify viral load (10) and the number of viral genome equivalent copies was calculated (E and ORF 1b gene: 10^6^ copy/μl, with 29 and 28 Ct, respectively).

### Isolation of SARS CoV-2 From Fecal Specimens

For the isolation of SARS-CoV-2 from fecal samples that tested positive for the virus in real-time RT-PCR analyses, will further process for isolation in Vero CCl-81 cell lines as per earlier published methods (11). The virus isolation experiment was conducted in a biosafety Level-4 facility at institute.

### Data Collection

Data were collected in the form of demographic information, epidemiological and clinical characteristics, which included medical history, comorbidities, signs and symptoms, laboratory investigations of all enrolled patients, from the record system of institute.

### Whole Genome Sequencing

The representative number (50) of fecal positive samples with the maximum viral load were selected for NGS analysis. The genome sequencing of SARS-CoV-2 was performed on the Ion AmpliSeq technology and the Ion Torrent personal genome machine (PGM). cDNA was synthesized with the SuperScript VILO reverse transcriptase kit (Invitrogen, USA) and the libraries were prepared according to the manufacturer's instructions (12).

After sequencing, data were analyzed with the complete genome of the SARS-CoV-2 Wuhan-Hu-1 isolate (GenBank accession number MN908947.3) using programs Bowtie 2 version 2.3.3.1 and Burrows-Wheeler Aligner (BWA) version bwa-0.5.9. All the sequences were submitted in GISAID database [EPI_ISL_1334317, EPI_ISL_1334318, EPI_ISL_1334319, EPI_ISL_1334320 EPI_ISL_1334321, EPI_ISL_1334322 EPI_ISL_1334323, EPI_ISL_1334324 EPI_ISL_1334325, EPI_ISL_7873234 EPI_ISL_7873235].

## Results

### Demographic and Baseline Data on the Basis of Fecal Sample Analysis

The demographics and clinical details of patients are shown in Table 1. The ratio of male to female patients with fecal samples positive for SARS-CoV-2 was 0.65:0.35 (182/98). The most cases were from age group of 31–70 years. The median age of positive patients with SARS-CoV-2 viral RNA was 47 years (range, 2–85 years). For patients whose fecal samples were positive and negative for SARS-CoV-2, the median time of intervals from disease onset to sampling was 9 days (range, 4–10 days).

**Table 1:** Demographics and baseline characteristics of enrolled patients.

**Gender**	
Male	182 (65%)
Female	98 (35%)
**Age median (range)**	Age Median (Range): 47 (2 Days-85 Years)
**Age group**	**Number (%)**
0–10	04 (1.43)
11–20	13 (4.64)
21–30	35 (12.50)
31–40	58 (20.71)
41–50	50 (17.86)
51–60	50 (17.86)
61–70	46 (16.43)
71–80	21 (7.50)
81–90	03 (1.07)

**Figure 1:**
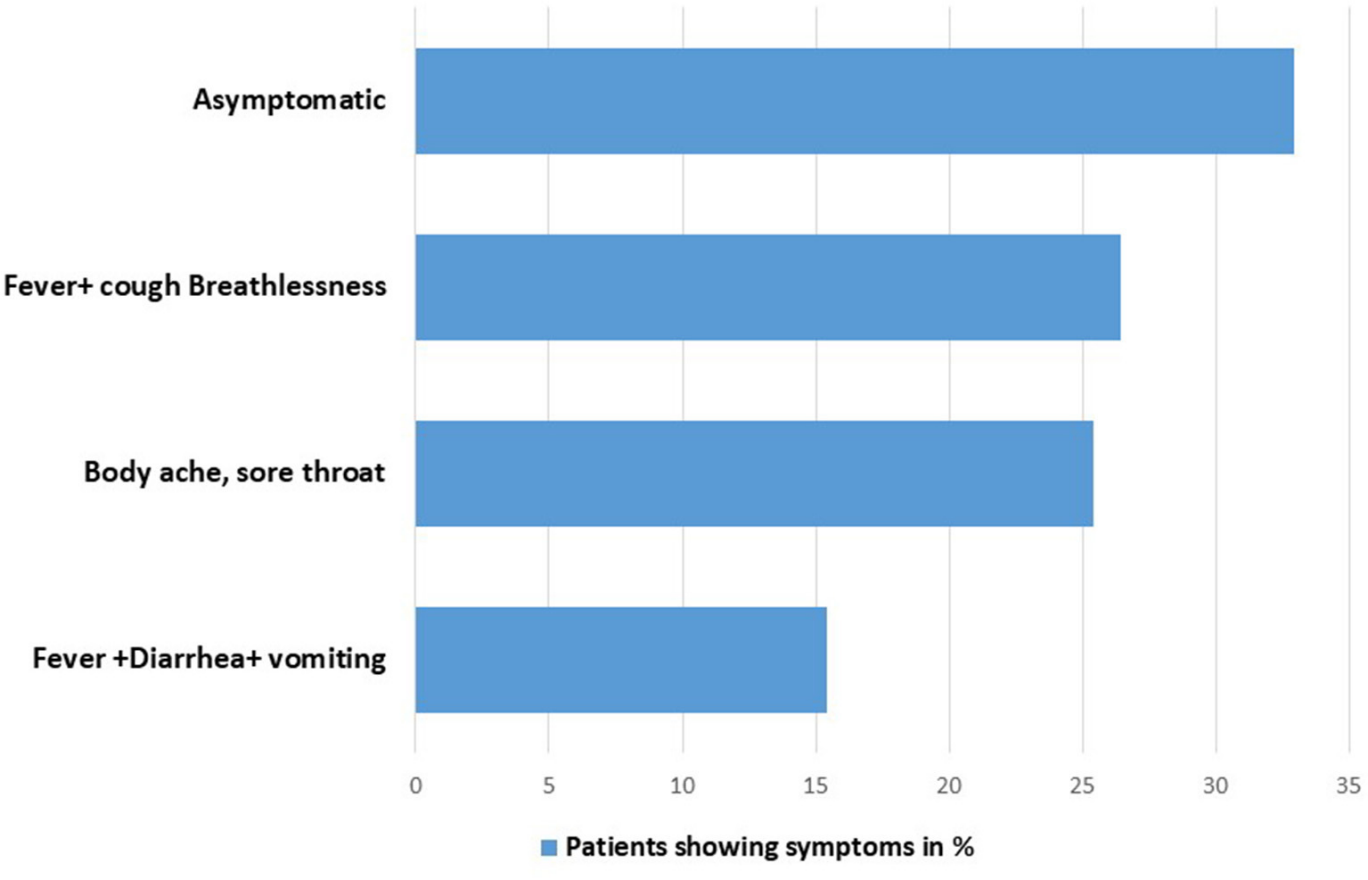
Symptomatic characteristics of enrolled patients.

Mostly, patients admitted in the hospital with the compliant of body ache, sore throat, fever, and cough hypoxia. Only 15.4% patients showed symptoms related to GI disorders, such as diarrhea and abdominal pain (Figure 1).

### Positive Rate of SARS-CoV-2 Nucleic Acid in Fecal Samples

**Figure 2:**
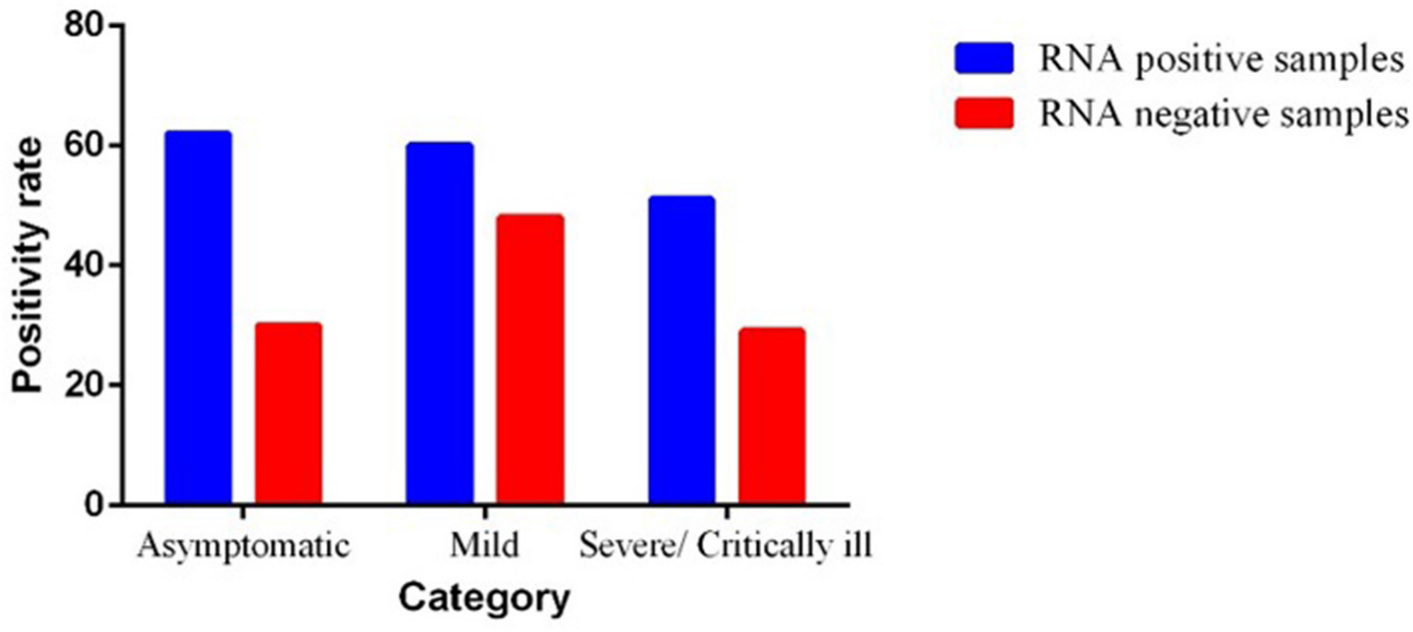
Severity of illness in severe acute respiratory syndrome coronavirus 2 (SARS-CoV-2) infected patients.

The results showed that fecal samples of 173 of 280 patients were positive for SARS-CoV-2 RNA, with a positive rate of 61.78%. Among them, the positive rate of SARS-CoV-2 RNA in fecal samples of critical/severe, moderate, and asymptomatic cases was 63.7% (51/80), 55.5% (60/108), and 67.39% (62/92), respectively. The Ct values in the fecal specimens were lower than in the throat specimens. We observed a significant difference (0.0061) between the PCR positive and negative groups in asymptomatic patients by using Mann–Whitney test (Figure 2).

### Severity of Illness in Patients With COVID-19

In total, 62 (67.39%) patients, who tested positive for SARS-CoV-2 RNA in feces, were identified as asymptomatic cases. A total of 108 (38.5%) patients belonged to mild cases, 60 of whom tested positive for viral RNA in feces and the other 48 cases tested negative. A total of 80 (28.57%) patients were categorized into severe cases who were having complaint of hypoxia, SPO_2_ <94%. Among them, 51 patients showed positivity for SARS-CoV-2 viral RNA and the other 29 patients tested negative in feces. The severity of SARS-CoV-2 infection is shown in Figure 2.

Data were analyzed by using Mann–Whitney test. However, there was no significant association among GI symptoms, severity, and fecal viral load. No significant difference was observed for the severity of illness between the two subgroups in severely ill patients. We observed the significant difference between the subgroups of asymptomatic and mild cases.

### Analysis of Viral Shedding Duration

**Figure 3:**
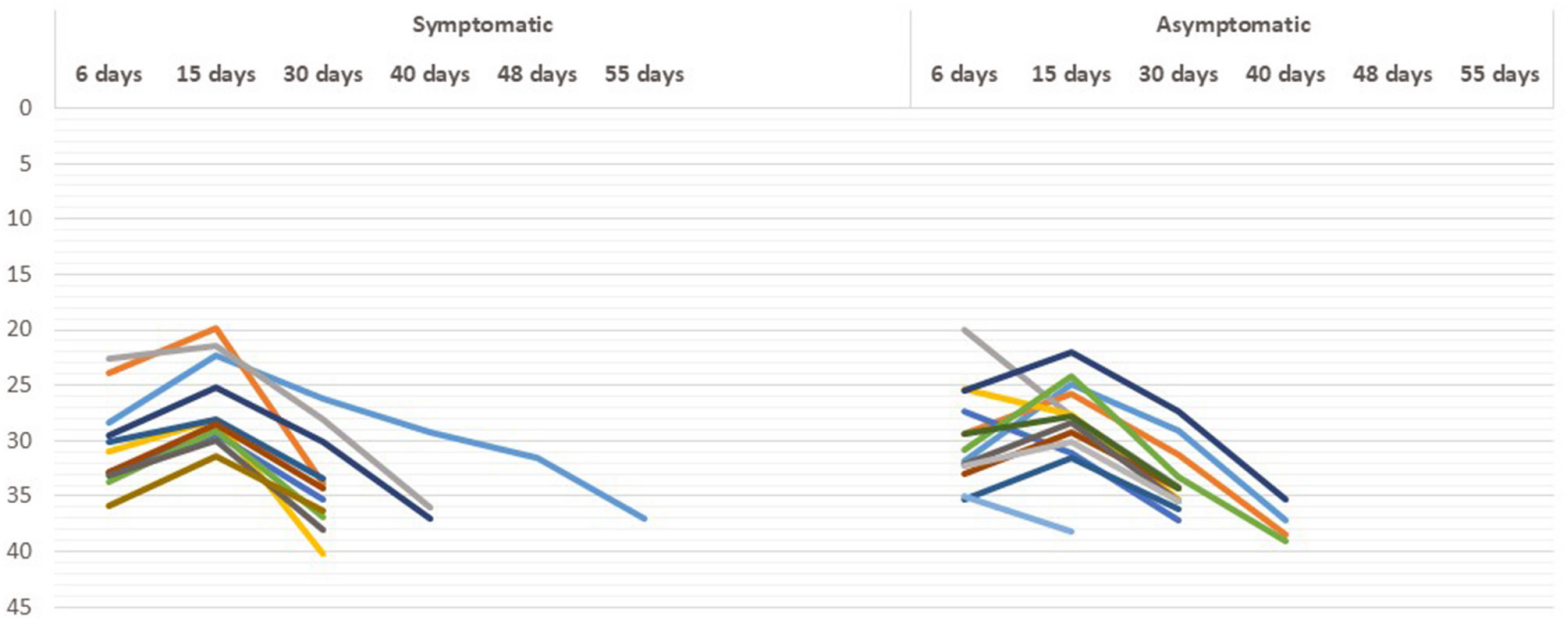
Real-time reverse transcription PCR (RT-PCR) results in asymptomatic and symptomatic individuals at different time period.

The time course of real-time RT-PCR test results for viral RNA in the fecal specimens of patients with COVID-19 during the hospitalization and recovery stage is shown in Figure 3. We observed that there was no significant difference in the time course of real-time RT-PCR negativity in both symptomatic and asymptomatic cases. The median (interquartile range [IQR]) duration time between onset of symptoms and the first positive RT-PCR test result for viral RNA was 11 (7–13) days in the fecal specimens.

**Figure 4:**
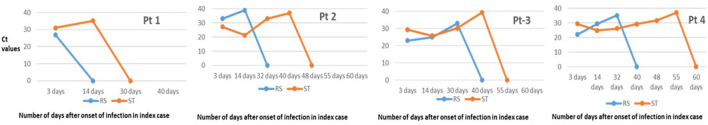
Chronologic changes in RT-PCR testing results.

We were able to investigate the chronologic changes in real-time RT-PCR test results in both fecal and respiratory specimens of the four patients only (Figure 4). No re-testing is recommended. This is due to guidelines from the Indian Government authorities regarding no re-testing prior to discharge from a COVID-19 facility after clinical recovery. In all the patients, the results of respiratory specimens were positive on day 32–40, fecal specimens converted to negative on day 30–55 [in pt 4] and the negative results were sustained on day 60.

### Phylogenetic Analysis of SARS-CoV-2 Sequence Obtained From Fecal Specimens From Positive Patients With COVID 19

**Figure 5:**
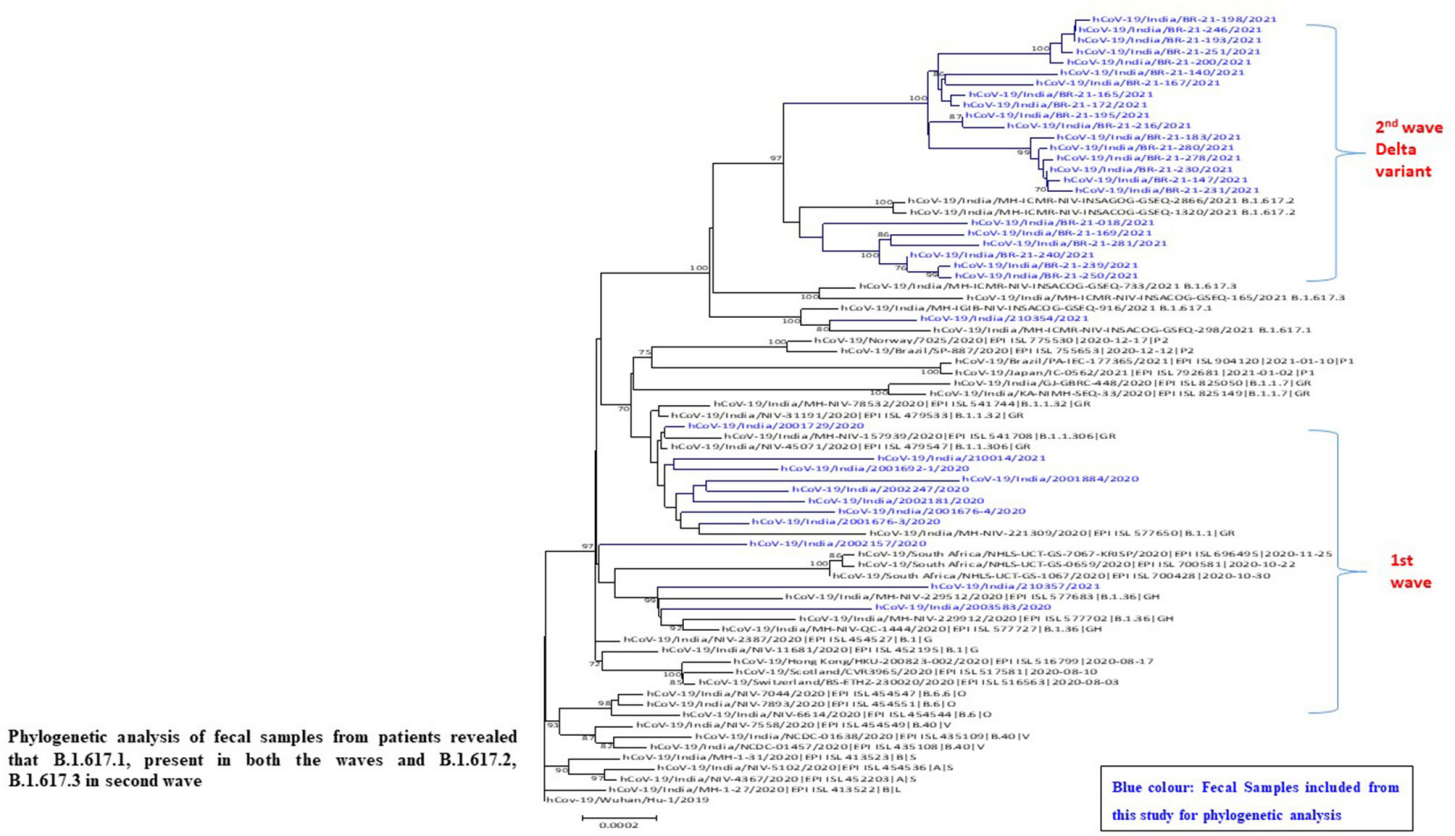
Phylogeny of SARS-CoV-2 genomes from fecal samples of SARS-CoV-2 positive patients from Pune, Maharashtra according to lineages.

For the further confirmation of presence of SARS CoV-2 in fecal samples, whole genome sequencing (*N* = 50) was performed from the representative number of fecal positive samples. We obtained complete consensus SARS-CoV-2 genomes from 35 of 50 samples (31%) processed for sequencing. NGS identified the whole genome sequence of SARS-CoV-2 from fecal specimens from positive patients with COVID-19 (confirmed oropharyngeal RT-PCR positive). During analysis, we observed one interesting finding that repeat sample of one patient (2001676-4) tested positive for SARS-CoV-2 by NGS from fecal even after 44 days of positive nasopharyngeal RT-PCR test. Phylogenetic analysis revealed that fecal samples from SARS-CoV-2 positive strains are a close congener with the strains mostly reported in the throat specimen from the same region (Figure 5).

### Analysis of Trend of Strains in Fecal Samples in Two Waves of SARS-CoV-2 Infection

Sequence analysis identified a number of nucleotide variants at position 241 [C-T], 3037 [C-T], 23403 [A-G], 14408 [C-T] across all positive patients and variants at positions 313 [C-T], 5700 [C-A], 28881–28882–28883. A number of silent mutations (241, 3037) and non-synonymous mutations (14408, 23403 and 28881–28882–28883) were also observed. The non-synonymous mutations were observed in ORF1b, ORF3a, and ORF8 (nucleocapsid phosphoprotein) genes, resulting in the amino acid mutation Q57H (glutamine to histidine), P227L (proline to leucine), and S194L (serine to leucine), respectively. D614G, one of the predominant mutation which is located in the spike glycoprotein was observed in all the sequenced genome in the first wave. One sample showed mutations at E154K, L452R, E484Q, P681R, Q1071H along with D614G which belongs to kappa variant (B.1.617.1 lineage).

Our results showed that the SARS-CoV-2 variants Alpha and Kappa were highly prevalent in the first wave of COVID-19 infection from the stool specimens also. In the second wave, the infection by B.1.617.2 (Delta) and B.1.617.3 (Figure 5) responsible for spreading the infection in second wave. Phylogenetic analysis of fecal samples from patients revealed that B.1.617.1 (Kappa) was present in both the waves.

### Isolation of Virus

We attempted culture of real-time PCR positive clinical samples multiple time from fecal specimens to understand infectivity. Fifty-five samples were taken for the isolation of SARS-CoV-2 based on Ct values. In addition, we amplified subgenomic RNA for E gene from these positive samples as a potential measure of replicating virus and observed Ct value in three of them. Virus isolation from fecal samples was not successful, irrespective of viral RNA concentration.

## Discussion

From the last 2 years, SARS-CoV-2 infection has spread and devastate the world at unusual rapid speed, emulating the 1918 flu pandemic. SARS-CoV-2, is highly contagious and stable in the environment and spreads mainly among humans through the respiratory route. To stop further spread of the infection, whole world is following the advice by the WHO which incorporates social distancing, self-isolation and quarantines or “lockdowns,” hand hygiene, and the use of private protective equipment (PPE) (3, 4). These current control measures are presumed on the understanding that SARS-CoV-2 infection is especially transmitted from infected persons *via* respiratory droplets during coughing and sneezing and through direct contact with infected persons and surfaces (3, 4). In our study, we found 62% from the fecal specimens of SARS-CoV-2 infected positive patients. The published literature suggest that SARS-CoV-2 may also be an enteric virus that can transmit through the fecal–oral route. Shedding of SARS-CoV-2 RNA in feces can be due to many reasons. SARS-CoV-2 entered through nose, eyes, and mouth to host cells with the help of angiotensin-converting enzyme-2 (ACE-2). ACE-2 has variable levels in GI tract, especially in esophagus, stomach, ileum, and colon (13). After entering through these receptors, SARS-CoV-2 enters the stomach. If the virus escapes gastric defenses, it could directly infect intestinal enterocytes and cause fecal shedding (7, 8). SARS-CoV-2 infected person sheds SARS-CoV-2 RNA for a mean duration of ~14–21 days, and the quantum of shedding ranges between 10^2^ and 10^8^ RNA copies per gram, but these viral load depends on the category of patients (14, 15). We analyzed similar observation in this study that the positive patients with complications or admitted in intensive care unit (ICU) were shedding virus for a longer duration (55 days) in comparison with asymptomatic/patients with mild symptoms (28–40 days). There was no significant difference in the mean duration of viral shedding between asymptomatic and symptomatic cases in GI tract. This raises the possibility of the fecal-oral route of transmission of SARS-CoV-2 in developing country where patients are asymptomatically infected and staying in crowded places, slums, etc. Such a hypothesis would also contribute to the rapidity and intensification of this pandemic.

Epidemiology of genomics uses NGS and high throughput sequencing of SARS-CoV-2 genomes to generate information related to recent transmission events, as well as the diversity of circulating variants in that particular region. By using the analyzed data from NGS of single nucleotide variants present in each sample, we detected the lineages present in that sample and compared them with lineages observed in Pune, India before to our sampling dates. SARS-CoV-2 sequence data generated from fecal samples indicates that there was a similar lineage circulating across the sampled communities. The study results indicating a shift in the SARS-CoV-2 sequence variation in fecal samples across the time. On November 26, 2021, the WHO distinguished the variant B.1.1.529 as a variation of concern, naming it Omicron, in the light of proof that Omicron contains various changes that might impact its behavior of transmissibility and vaccine effectiveness. A recent study done by Kumar et al. (16) observed that the Omicron variants had a higher fondness for human angiotensin-changing over catalyst 2 (ACE2) than the Delta variation because of countless transformations in the SARS-CoV-2 receptor-restricting area (RBD), showing a higher potential for transmission by using computational approaches.

The limitations in our study was that some patients were uncomfortable and reluctant to participate in a study that involved the collection of stool samples. As well as after getting discharge from hospitals, it was difficult to trace the patients for repeat samples to check the shedding of virus. SARS-CoV-2 is capable of infecting the GI tract and shedding *via* fecal samples of SARS-CoV-2 infected individuals within the environment could be playing a possible route from human-to-human transmission. From both clinical and public health view, it is vital to completely understand the route of transmission of SARS-CoV-2. As per in reference with public health issue, viral particles within the feces shed by infected individuals, if aerosolized, have great involvement in compact environments, such as cruise ships, hospitals, individual households, and densely populated housing, especially in regions with poor sanitation. There are several potential risk factors and risky practices predisposing human health to risks in developing countries, such as poor wastewater management, poor sanitation and hygiene, high risk of co-infections, and lack of surveillance systems, promoting the SARS-CoV-2 fecal-oral transmission. These risk factors and poor practices could also be one among the likelihood for fecal-oral transmission and therefore the related adverse human health outcomes might be apparently higher in developing than developed countries. To regulate the pandemic, every effort should be made to know all the possible routes of transmission of the infections, even the smaller ones.

## Data Availability Statement

The datasets presented in this study can be found in online repositories. The names of the repository/repositories and accession number(s) can be found in the article/supplementary material.

## Ethics Statement

The studies involving human participants were reviewed and approved by Institutional Ethics Committee, ICMR-National Institute of Virology. Written informed consent to participate in this study was provided by the participants or their legal guardian/next of kin.

## Author Contributions

ML, SR, and VP contributed in the conception of the work and experimentation was done by ML, SR, MJ, MS, NC, PMS, and ST. SM and PS did the isolation work. ML and SJ did the bioinformatics analysis. VP, SP, and RK contributed in recruiting the patients. ML and MJ: analysis and manuscript writing. All authors contributed to the article and approved the submitted version.

## Funding

This study was funded by the ICMR-National Institute of Virology, Pune, India and Indian Council of Medical Research, New Delhi, India.

## Conflict of Interest

The authors declare that the research was conducted in the absence of any commercial or financial relationships that could be construed as a potential conflict of interest.

## Publisher's Note

All claims expressed in this article are solely those of the authors and do not necessarily represent those of their affiliated organizations, or those of the publisher, the editors and the reviewers. Any product that may be evaluated in this article, or claim that may be made by its manufacturer, is not guaranteed or endorsed by the publisher.
